# A Prospective Observational Cohort Study on Orthopaedic and Anaesthetic Registrars Performing Femoral Nerve Block on Patients with an Acute Hip Fracture

**DOI:** 10.1155/2016/7512360

**Published:** 2016-09-15

**Authors:** Åsa Thelaus, Tobias Pettersson, Max Gordon, Ferid Krupic, Olof Sköldenberg

**Affiliations:** ^1^Division of Orthopaedics, Department of Clinical Sciences at Danderyd Hospital, Karolinska Institutet, Stockholm, Sweden; ^2^Division of Anesthesiology, Department of Clinical Sciences at Danderyd Hospital, Karolinska Institutet, Stockholm, Sweden; ^3^Department of Orthopaedics, Institute of Clinical Sciences, Sahlgrenska Academy, University of Gothenburg, Gothenburg, Sweden

## Abstract

We investigated if a femoral nerve block (FNB) for patients with a proximal femoral fracture (PFF) and administered by an orthopaedic registrar (OR) instead of an anaesthesiology registrar (AR) lowers the lead time to block and reduces the total amount of rescue analgesics during the preoperative phase. 205 patients were included in a prospective observational cohort study. The main outcome variable was rescue analgesics as total intravenous morphine prior to surgery. All results were adjusted for confounding using age, sex, cognitive dysfunction, and ASA classification. The OR group (*n* = 135) was over 2 hours faster in performing the block compared to the AR group (*n* = 70) but was nonetheless correlated with an increased amount of rescue analgesics during the study, 2.4 mg morphine (95% CI 0.0–4.9) more compared to the AR group. We found no difference between the groups in the risk of adverse events. We conclude that, for patients with an acute PFF and with morphine consumption as end point, how soon from arrival to hospital the patients receive a FNB is of lesser importance than who is administering it. Based on our results we recommend that emergency hospitals should have routines for anaesthesiologists performing FNB on this frail patient group.

## 1. Introduction

Proximal femoral fractures (PFF) including the femoral neck and inter- and subtrochanteric fractures are among the most common, and in Sweden, more than 18,000 patients with femoral proximal femur fractures (PFF) are operated on annually [1]. In many hospitals, fast tracking of PFF patients is used to operate on patients within 24 hours from arrival [1]. During the preoperative phase the patient goes through several potentially painful manoeuvres such as moving from one bed to another, change of clothes, and shower. A femoral nerve block (FNB) is an effective way of reducing pain in patients with PFF [2, 3]. The routines for administration of FNB vary between hospitals and can be administered either by an anaesthesiologist along with preoperative examination or an orthopaedic surgeon/registrar at the emergency room. As the availability of anaesthesiologist can vary between hospitals and care-settings, training orthopaedic registrars to administer FNBs immediately at admission is an appealing alternative.

The aim of the study was to investigate if a FNB received early upon the arrival to the hospital, administered by an orthopaedic registrar, lowers the total amount of morphine given to patients during the preoperative phase, compared to anaesthesiology registrars.

## 2. Material and Methods

### 2.1. Study Design and Setting

We performed a prospective observational cohort study between May 2012 and June 2013 at the Danderyd Hospital, Stockholm, Sweden. Danderyd hospital is a university teaching hospital and has a catchment area of approximately 500,000 inhabitants. The guidelines of STROBE [4] statement were followed and ethical approval was granted from the regional ethical review board.

### 2.2. Subjects and Eligibility Criteria

We consecutively included patients arriving to the hospital during the study period. The inclusion criterion was an X-ray verified PFF (femoral neck/trochanteric/subtrochanteric). We excluded patients with multiple fractures and those with infection in the injection site or presence of a prosthetic femoral artery graft or any other condition that was considered a contraindication for FNB [2, 3]. The patients were divided into two groups, depending on who administered the FNB, orthopaedic (OR) or anaesthesiology registrars (AR). The choice of who administered the FNB (OR or AR) was done randomly by availability of specialty doctor when the patient arrived. No formal allocation sequence was used.

### 2.3. Variables

#### 2.3.1. Data Collection

Two of the authors (Åsa Thelaus and Tobias Pettersson) reviewed the patients' charts and constructed a digital case-report form that was used throughout the study. Data was gathered for how much morphine was given: before admission, after admission before the FNB, and 10 h (or until time of surgery if it was <10 h) after the FNB. All the opiates were recalculated into morphine iv equivalents [5]. We also reviewed the charts for adverse events to morphine and if any antiemetics were administered.

#### 2.3.2. Outcomes and Exposure

The primary outcome was the amount of administered iv morphine during the preoperative phase. This was measured from the time of arrival of the patient at the hospital until 10 h after FNB, or until time of surgery if <10 h. The secondary outcome measures were time from arrival at hospital to FNB, iv morphine in the two groups after FNB, and adverse events to morphine, that is, nausea and vomiting requiring administration of antiemetics. The exposure was defined according to who administered the FNB, AR (*n* = 7) or OR (*n* = 10).

#### 2.3.3. Confounders

Confounders are variables with a potential impact on both exposure and outcome variables. We used a Directed Acyclic Graph to identify potential confounders [6]. The confounders adjusted for were age, sex, ASA classification, and cognitive dysfunction.

### 2.4. Femoral Nerve Block

The routine for easing pain at our hospital consists of paracetamol 665 mg, oxycontin 5–20 mg, oral morphine 10 mg, and iv morphine given as a rescue analgesia, dose given until VAS ≤ 4. Pain ascending from a proximal femur fracture can be reflected to be produced by a combination of the fractured bone itself, stretching of the joint capsule, and spasm of the surrounding muscles [7]. If unsatisfactory pain relief (Visual Analouge Scale [VAS] > 4) was found, a FNB was administered using a Stimuplex® needle inserted after locating specific landmarks [8]. The patient is placed supine with leg extended. The inguinal ligament is identified and a line is drawn between the anterior superior iliac spine and the pubic symphysis. The femoral pulse is palpated and marked. The needle is placed 2 cm laterally and 2 cm distally of that mark. The pulse frequency of the nerve stimulator is set to 2 Hz and the pulse width to 100 *μ*s and the output to 2 mA. After receiving appropriate motor response, twitching of the quadriceps muscle, the output is tuned down to 0.6 mA and if the motor response is sustained but disappears below 0.2 mA the local anaesthetic, 20 mL ropivacain 7.5 mg/mL, is given (aspirating every 5 mL, reassuring not placing the local anaesthetic intravenously). The efficacy of the neural blockade was evaluated after 10 minutes by testing for cold using a sponge with alcohol on the skin.

Prior to the start of the study period the OR and AR group were given a theoretic lecture literature to read about anatomy of the area and a description of the procedure of administering a FNB. They also performed a hands-on trial, where the anatomy was demonstrated with ultrasound and then they administered a FNB on the main author (Åsa Thelaus) with the ropivacain exchanged with NaCl. A cart was set up at the orthopaedic ward with all necessary equipment for administering a FNB with a text document and photos describing the procedure attached to the cart.

### 2.5. Sample Size

Prior to the start of the study, we conducted a power analysis (two-sided, *p* = 0.05) and tested the null hypothesis that the mean value of the total administered iv morphine (in morphine equivalents) and the proportion of adverse events of opiates would be equal in both groups. Based on previous published papers on FNB, a mean difference in morphine consumption of 5 mg (SD 6) and a 20% difference (20% OFNB and 40% AFNB ) in the rate of negative side effects were, in our opinion, the smallest effect sizes that would be clinically relevant [7]. From a small pilot series at our department, we knew that approximately 2/3 of patients were given the FNB by an orthopaedic registrar. We calculated that a total of 60 patients (40 OR and 20 in AR group) would have a power of 80% to yield a statistically significant result for morphine consumption. We also calculated that 180 patients (120 in OR and 60 in AR group) would have a power of 80% to yield a statistically significant result for adverse events. We therefore planned to include at least 200 patients to account for loss of data.

### 2.6. Statistics

We used linear regression for administered morphine and time to FNB outcomes and logistic regression for adverse events outcome. Each model used age, sex, and ASA classification as confounders and the exposure variable (AR or OR group) in the analysis. The analyses were performed using R 3.2.2, using the rms-package (v. 4.2-1) for modeling, knitr (v. 1.7) for reproducible research, and Gmisc (v. 11) with Greg (v. 1.1.0) for table output.

## 3. Results

### 3.1. Participants and Descriptive Data

We screened 299 patients and included 205 patients during the 13-month study period, 135 in the OR group and 70 in the AR group. 94 patients were excluded since they did not receive a FNB. The baseline characteristics of the two groups were similar (Table 1). There were no complications reported for any FNB and the FNB resulted in neural blockade in all patients.

### 3.2. Morphine Consumption

FNB in the OR group was correlated with an increased amount of administered morphine during the study, 2.4 mg (95% CI 0.0–4.9) after adjustments. Out of the other confounding variables in the model, increasing age and the presence of cognitive dysfunction lowered the amount of administered morphine (Table 2). We also found that patients in the OR group received 1.4 mg (95% CI 0.3–2.7) more morphine after the FNB, indicating a poorer effect of the FNB (Tables 1 and 2).

### 3.3. Time to Nerve Block and Adverse Events

The OR group was approximately 2 hours faster in administering the FNB, −2.2 hours (95% CI −2.9–−1.6). In addition, an ASA class of 3-4 was correlated with a slight delay to FNB compared to ASA 1-2, −0.3 hours (95% CI −0.5–0.0) (Tables 1 and 2). A total of 50 patients (24%) experienced adverse events in the form of nausea and/or vomiting during the preoperative phase. We found no difference between the groups in the risk of adverse events. However, male sex was correlated with a significantly lower risk compared to females of adverse events, odds ratio 0.3 (95% CI 0.1–0.8).

## 4. Discussion

In this prospective observational cohort study on patients with a PFF and FNBs given by either an anaesthesiology or orthopaedic registrar, we found that FNB administered by an OR is correlated with increased need for morphine before surgery. This was found despite the fact that the OR group was faster in performing the block when the patients arrived at the hospital.

### 4.1. Strengths

The key strength in our study is the sample size, based on a prestudy sample size estimation, and complete follow-up. We also included a representative sample of elderly PFF patients with comorbidities and cognitive dysfunction with similar characteristics as previous studies on hip-fracture patients [1, 9, 10].

### 4.2. Limitations

Our main limitation is the lack of formal randomization. Allocation to the two groups was performed by what specialty randomly had a registrar available for the FNB but we cannot rule out selection bias in the exposure variable. A patient with pain may spur an OR towards performing the block despite having to take care of other obligations. Conversely a patient with little pain may be perceived as more acceptable to receive their FNB later. Furthermore the amount of intravenous morphine is dependent on the nurse's perception of the patient. It is plausible that patients with more pain will prompt the orthopaedic nurse to contact her nearest doctor, the orthopaedic registrar, for help. Thereby again selecting the patients who are in more pain for intervention. In addition, the VAS > 4 for pain was only used in the study when selecting which patients needed a FNB. This was chosen since a large proportion of our patients had cognitive dysfunction and we anticipated that we could not get reliable data on repeated VAS scoring on these patients. The study was performed during the introduction of the new routine of the ORs administering the FNB and includes thus the learning curve.

Based on our findings, orthopaedic registrars do not administer the FNB as efficiently as anaesthesiology registrars. This is surprising as the OR group received their FNB more than 2 hours earlier than the AR group.

In previous studies with patient reported pain score (VAS) the FNB gave significantly faster pain relief and a greater reduction of VAS than iv morphine [7, 11]. Those studies did not however include patients with cognitive dysfunction. In another study by Newman et al. [8] the authors compared fascia iliaca block (FIB) versus the 3 in 1 block FNB for femoral neck fractures and the author showed that a FNB was superior to FIB. In this study, 93% of the blocks were delivered by two operators and the difference between the means of VAS score reduction was 0.9 cm (95% CI 0 to 1.8 cm *p* = 0.04). A recent (2014) survey in the UK indicated that only 44% of emergency hospitals routinely performed any form of regional anaesthesia to PFF patents. The most commonly used method was the FNB (60% of blocks) followed by FIB [12]. For Sweden there are no statistics available on the use of FNB or FIB, indicating that there is room for improvement in preoperative care for hip fractured patients.

We also found that patients with cognitive dysfunction received significantly lower rescue opioids. Previous studies have demonstrated the difficulties of caring and communicating with this patient group in the perioperative phase [13]. We believe that patients with cognitive dysfunction may have a harder time communicating pain and therefore benefit from a standardized procedure as FNB rather than depending on rescue analgesia, where dose and time of administration require the ability to communicate insufficient pain relief.

We performed our study in the clinical setting of a medium sized emergency hospital, we had wide inclusion and narrow exclusion criteria. A large proportion (69%) of screened patients were included in the study and we therefore believe that our results reflect the reality of everyday work in an orthopaedic unit.

## 5. Conclusion

We conclude that, for patients with an acute PFF and with morphine consumption as endpoint, how soon from arrival to hospital the patient receives a FNB is of lesser importance than who is administering it. Based on our results we recommend that emergency hospitals should have routines for anaesthesiologists performing FNB on this frail patient group.

## Figures and Tables

**Table 1:** Characteristics of the patients and main outcomes.

	AR group (*n* = 70)	OR group (*n* = 135)
*Baseline*		
Age (years)^1^	82 (10)	83 (9)
Sex^2^		
Female	57 (81%)	96 (71%)
Male	13 (19%)	39 (29%)
Weight (kg)^2^	63 (13)	66 (13)
ASA classification^2^		
1-2	19 (27%)	43 (32%)
3-4	51 (73%)	92 (68%)
Cognitive dysfunction^2^		
No	47 (67%)	85 (63%)
Yes	23 (33%)	50 (37%)
Type of fracture^2^		
Femoral neck	35 (50%)	61 (45%)
Trochanteric	31 (44%)	63 (47%)
Subtrochanteric	4 (6%)	11 (8%)
*Outcomes*		
Total morphine consumption (mg)^1^	11.7 (8.8)	13.6 (8.5)
Adverse events^2^		
No	50 (71%)	105 (78%)
Yes	20 (29%)	30 (22%)
Time from arrival to FNB (h)^1^	5.3 (3.0)	3.0 (1.9)
Morphine consumption before FNB (mg)^1^	9.0 (7.9)	9.5 (6.5)
Morphine consumption after FNB (mg)^1^	2.7 (3.9)	4.1 (5.0)

^1^Mean (SD)

^2^ *n* (%).

**Table 2:** Linear and logistic regression analyses with crude and adjusted outcomes. Potential confounders adjusted for are age, sex, ASA class, and cognitive dysfunction. Only the exposure variable (group) and confounders with *p* values ≤ 0.05 are presented.

	Crude		Adjusted	
	Est.	95% CI	Est.	95% CI
*Linear regression (per unit)*				
*Total morphine consumption (mg)*				
OR group	1.9	−0.6–4.4	2.4	0.0–4.9
Age	−0.3	−0.4–−0.2	−0.3	−0.4–−0.1
Cognitive dysfunction	−3.8	−6.2–−1.3	−2.8	−5.3–−0.2
*Time to FNB from arrival (h)*				
OR group	−2.2	−2.9–−1.5	−2.2	−2.9–−1.6
ASA 3-4	0.5	−0.3–1.3	0.3	−0.5–1.0

*Logistic regression (odds ratio)*				
*Adverse event*				
OR group	0.7	0.4–1.4	0.8	0.4–1.5
Male sex	0.3	0.1–0.7	0.3	0.1–0.8
